# Extremely low-frequency phonon material and its temperature- and photo-induced switching effects†

**DOI:** 10.1039/d0sc02605k

**Published:** 2020-08-17

**Authors:** Takaya Yoshida, Koji Nakabayashi, Hiroko Tokoro, Marie Yoshikiyo, Asuka Namai, Kenta Imoto, Kouji Chiba, Shin-ichi Ohkoshi

**Affiliations:** Department of Chemistry, School of Science, The University of Tokyo 7-3-1 Hongo, Bunkyo-ku Tokyo 113-0033 Japan ohkoshi@chem.s.u-tokyo.ac.jp; Division of Materials Science, Faculty of Pure and Applied Sciences, University of Tsukuba 1-1-1 Tennodai Tsukuba Ibaraki 305-8573 Japan; Material Science Div., MOLSIS Inc. Tokyo Daia Bldg., 1-28-38 Shinkawa, Chuo-ku Tokyo 104-0033 Japan

## Abstract

Atomic vibrations due to stretching or bending modes cause optical phonon modes in the solid phase. These optical phonon modes typically lie in the frequency range of 10^2^ to 10^4^ cm^−1^. How much can the frequency of optical phonon modes be lowered? Herein we show an extremely low-frequency optical phonon mode of 19 cm^−1^ (0.58 THz) in a Rb-intercalated two-dimensional cyanide-bridged Co–W bimetal assembly. This ultralow frequency is attributed to a millefeuille-like structure where Rb ions are very softly sandwiched between the two-dimensional metal–organic framework, and the Rb ions slowly vibrate between the layers. Furthermore, we demonstrate temperature-induced and photo-induced switching of this low-frequency phonon mode. Such an external-stimulation-controllable sub-terahertz (sub-THz) phonon crystal, which has not been reported before, should be useful in devices and absorbers for high-speed wireless communications such as beyond 5G or THz communication systems.

## Abstract 2

Extremely low-frequency optical phonon mode in Rb-intercalated two-dimensional cyanide-bridged Co–W bimetal assembly and its temperature- and photo-induced switching effect.

## Graphical abstract

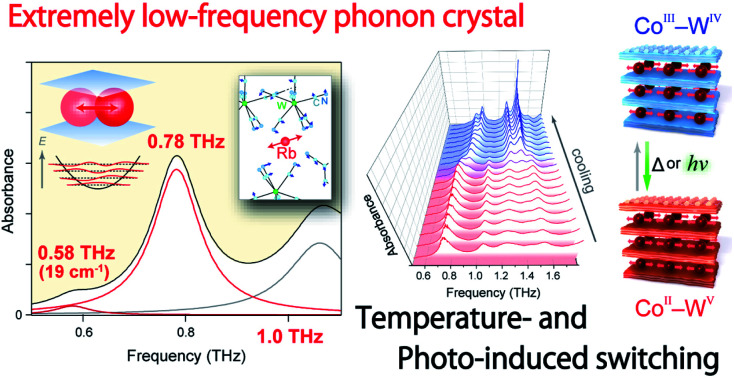

## Introduction

**Fig. 1:**
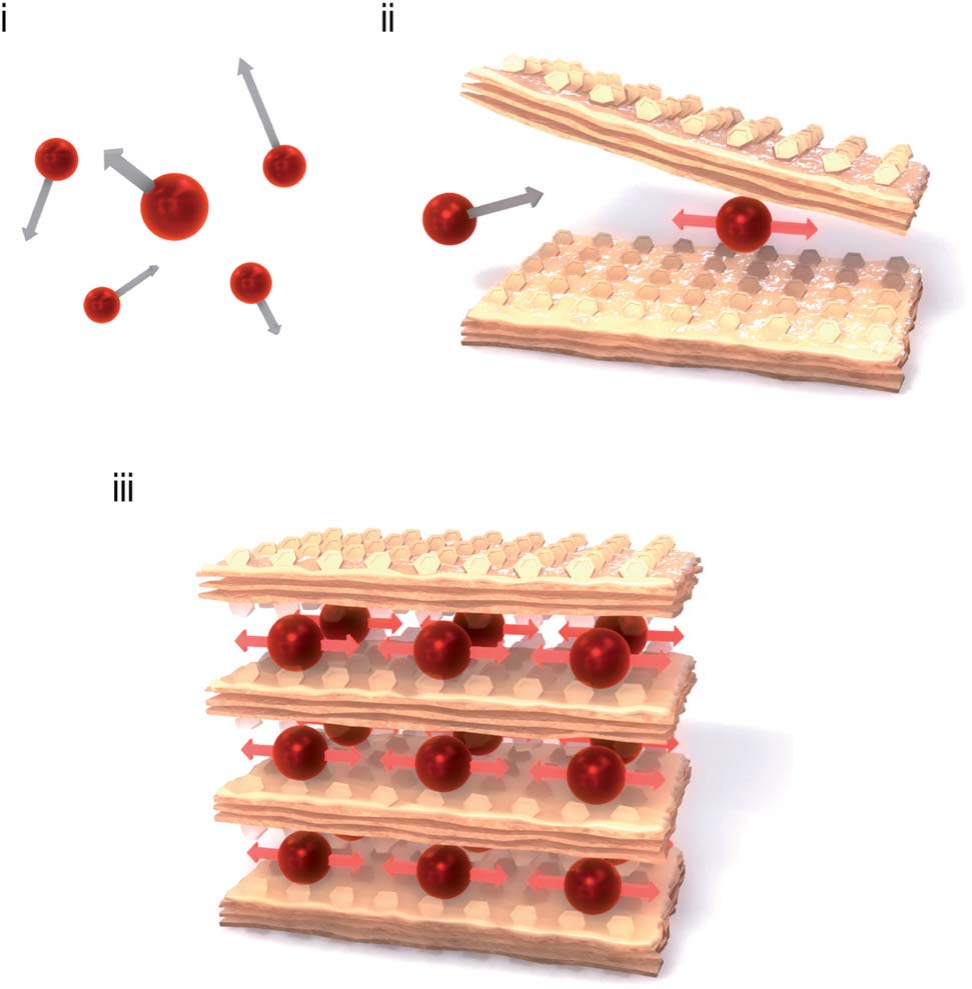
Strategy to construct an extremely low-frequency optical phonon mode. (i) Monoatomic molecules in the gas phase or liquid phase move with free motion (translational mode). (ii) If a monoatomic molecule is caught by a two-dimensional (2D) metal–organic soft framework, the translational mode switches to a vibrational mode with a low vibrational frequency. (iii) The material in the present work, that is, a 2D layered metal–organic framework with heavy atoms which are lightly enfolded by the soft framework with organic ligands.

A monoatomic molecule in the gas or liquid phase moves with free motion. Such a movement of a monomolecule is called a translational mode (Fig. 1(i)). By contrast, bonded atoms in a molecule or a solid phase vibrate with other atoms coherently, which is called the vibrational mode. The vibrational motion is observed as the optical phonon mode.^1–7^ Typical stretching vibrational modes are observed at several tens of terahertz (THz), *e.g.*, CO stretching mode (2143 cm^−1^, 64 THz) and CN stretching mode (2200 cm^−1^, 66 THz). If a heavy monoatomic molecule is softly caught by an organic cage or a metal–organic framework cage (Fig. 1(ii)), the translational mode switches to a vibrational mode. The vibrational frequency of the trapped monoatom should be lower than that of a typical bonded atom. To design a low-frequency optical phonon mode, the size and shape of the cage and the chemical affinity of the surrounding framework are both important. Recently, a Cs^+^ ion encapsulated in a three-dimensional (3D) –M–C≡N–M′– framework of a Prussian blue analogue showed a low-frequency vibrational mode of 1.4 THz.^8^ In this case, the Cs^+^ ion is surrounded by twelve –C≡N– anionic sides of the cubic lattice. By contrast, the number of sides in a two-dimensional (2D) –M–C≡N–M′– framework decreases to eight. Thus, the frequency of the vibrational mode should be lower. As shown in Fig. 1(iii), a monoatom in a 2D framework should vibrate at a lower frequency (Movie S1†) compared to that in a hollow box of a 3D framework. From this perspective, soft frameworks based on 2D metal–organic frameworks and coordination polymers^9–24^ such as cyanide-bridged bimetal assemblies^25–40^ are strong candidates. The low-frequency vibrational mode (optical phonon) is very attractive from the viewpoint of THz technology.^41–43^ To date, several examples of low-frequency phonon modes have been reported.^44,45^ Because sub-THz light-resonating materials have potential as optical devices and absorbers for next-generation wireless communications, *i.e.*, millimetre wave communication^46–48^ or beyond 5G communication,^49^ they will take an important role in the era of big data and the Internet of Things (IoT).^50,51^ Additionally, from the viewpoint of thermoelectrics, low-frequency optical phonon modes due to atomic rattlers have drawn attention.^52–55^

In the present work, we synthesise a Rb-intercalated 2D cyanide-bridged Co–W bimetal assembly, Rb^I^[Co^II^(3-cyanopyridine)_2_][W^V^(CN)_8_] (**RbCoW**), and investigate its optical phonon mode by THz time-domain spectroscopy (THz-TDS). Additionally, its temperature- and photo-induced switching effects are examined from the viewpoint of dynamic functionalities.

## Results and discussion

### Material and crystal structure

A single crystal of **RbCoW** was obtained by the slow diffusion method in an H-shaped tube. An aqueous solution of cobalt dichloride hexahydrate, 3-cyanopyridine, and rubidium chloride was diffusively mixed with an aqueous solution of rubidium octacyanidetungstate hydrate and rubidium chloride. After one month, a crystal was obtained. The powder-form sample was obtained by mixing a solution of cobalt dichloride, 3-cyanopyridine, rubidium chloride, and rubidium octacyanidetungstate with a magnetic stirrer for one day. The precipitates were collected and placed under vacuum conditions at room temperature. Inductively coupled plasma mass spectrometry and elemental analysis showed that the formula is Rb^I^[Co^II^(3-cyanopyridine)_2_][W^V^(CN)_8_] (see Methods).

**Fig. 2:**
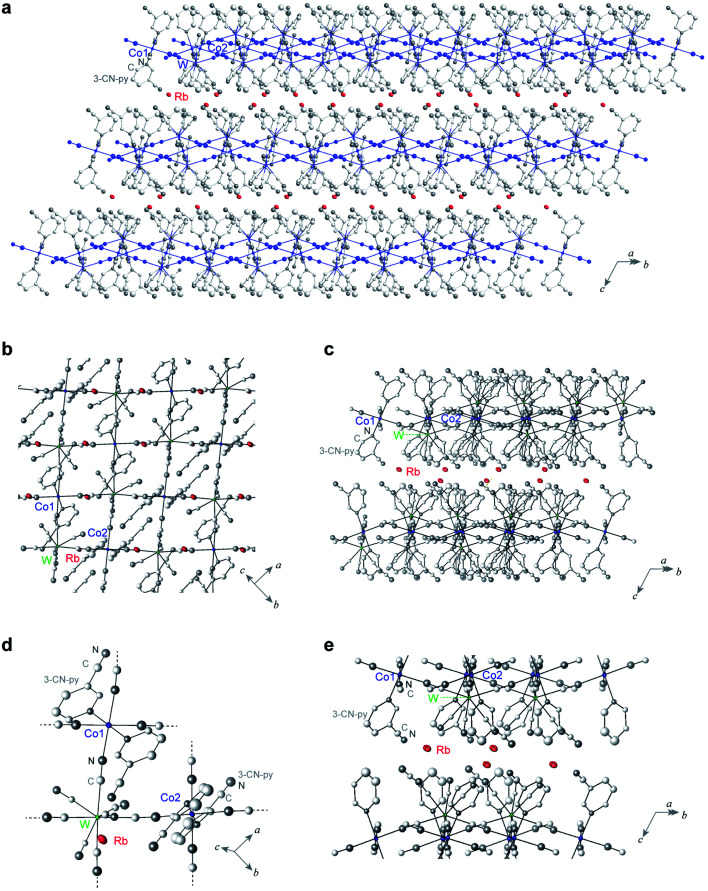
Crystal structure of **RbCoW**. (a) Rb-intercalated 2D cyanide-bridged Co–W bimetal assembly. Red ellipsoids indicate the Rb ions. Co, W, C, and N atoms forming the 2D network are shown as blue ellipsoids and balls, whereas the other C and N atoms are represented as light grey and grey balls. (b) Projection of the 2D network layer in the *ab*-plane. (c) Side view of the layers. (d) Coordination structure of **RbCoW**. (e) Structural environment around the Rb ions between the layers. Red, blue, and green ellipsoids represent Rb, Co, and W atoms, and light grey and grey balls represent C and N atoms, respectively. Displacement ellipsoids and balls are drawn at the 30% probability level. H atoms are omitted for clarity.

Single-crystal X-ray analyses revealed that the crystal structure of **RbCoW** at 300 K is a triclinic system in the *P*1̄ space group with cell parameters of *a* = 7.5298(4) Å, *b* = 13.7545(7) Å, *c* = 14.0687(6) Å, *α* = 119.276(4)°, *β* = 101.020(6)°, *γ* = 89.974(6)°, and *V* = 1240.15(12) Å^3^ (Fig. 2 and Table S1†). The Co–W bridged by cyanides forms 2D layers in the *ab*-plane, which are stacked along the *c*-axis and involves the Rb ions between the layers. The asymmetric unit consists of two halves of Co ions, a [W(CN)_8_] ion, two 3-cyanopyridine molecules, and a Rb ion. The coordination geometry of [W(CN)_8_] ion is close to a bicapped trigonal prism (*C*_2v_). Four cyanide ligands of the [W(CN)_8_] ion are coordinated to the nearest neighbouring Co ions, and the other four cyanide ligands are free. The coordination geometry of the Co ion is a six-coordinated pseudo-octahedron (*D*_4h_). The two axial positions of the Co ion are occupied by the nitrogen atoms of two 3-cyanopyridine molecules, while the cyanide nitrogen atoms of [W(CN)_8_] ion are in the four equatorial positions.

### First-principles phonon mode calculations

**Fig. 3:**
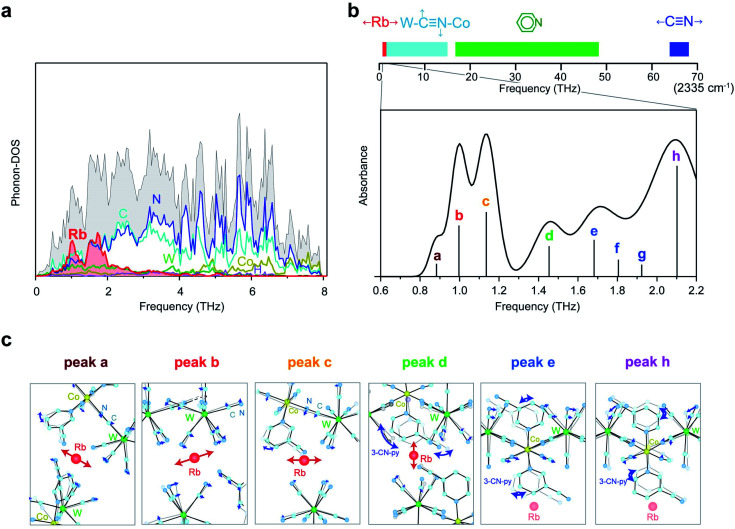
First-principles phonon mode calculations. (a) Phonon density of states (phonon-DOS) of **RbCoW**. Black, red, dark yellow, green, light blue, blue, and purple lines indicate the total phonon-DOS and the partial phonon-DOS of Rb, Co, W, C, N, and H, respectively. (b) Upper part is the frequency bar in the rage of 0–70 THz (0–2335 cm^−1^) indicating the phonon modes of **RbCoW**. Red, light blue, green, and blue bars represent the frequency regions of the Rb vibration, transverse translational and transverse librational modes of the CN ligands, phonon modes of the pyridine ligands, and CN stretching modes, respectively. The low-frequency region of 0.6–2.2 THz is enlarged to show the optical phonon spectrum calculated from the IR active phonon modes. (c) Atomic movements of the phonon modes corresponding to peaks a–e and h. Peaks a–c are assigned to the phonon modes of Rb ion vibrations in the *ab*-plane. Atomic movement of peak d is due to Rb ion vibrations along the crystallographic *c*-axis and 3-cyanopyridine ligand rotation. Peak e is attributed to the combination of transverse translational modes of W–CN–Co and 3-cyanopyridine ligand rotation, and peak h originates from the transverse librational modes of W–CN–Co and 3-cyanopyridine ligand rotation.

To estimate the frequency of the optical phonon modes of **RbCoW**, the phonon modes were calculated by the Phonon code from the determined crystal structure and atomic positions (Tables S2–S4,† Methods). The phonon density of states (phonon-DOS) is shown in Fig. 3a and S1.† Additionally, the optical phonon spectrum was calculated based on the transition probabilities of the vibrational modes. The calculated optical phonon mode spectrum shows that **RbCoW** has eight absorptions in the THz region, *i.e.*, peaks at 0.883 (peak a), 0.997 (peak b), 1.136 (peak c), 1.453 (peak d), 1.682 (peak e), 1.805 (peak f), 1.923 (peak g), and 2.102 THz (peak h) (Fig. 3b). From the video of the atomic movements (Movie S2†), peaks a–c are assigned to the phonon modes of Rb ion vibrations in the *ab*-plane, while peak d is mainly due to Rb ion vibrations along the crystallographic *c*-axis. In these phonon modes, the 3-cyanopyridine and cyanide ligands sway together with the Rb ions, supporting the Rb movements. Peaks e and f are attributed to a combination of transverse translational modes of W–CN–Co and 3-cyanopyridine ligand rotation, while peaks g and h originate from the transverse librational modes of W–CN–Co and 3-cyanopyridine ligand rotation. Fig. 3c shows the atomic movements of the intense absorption peaks a–e, and h.

### Optical phonon mode measurements by THz-TDS

**Fig. 4:**
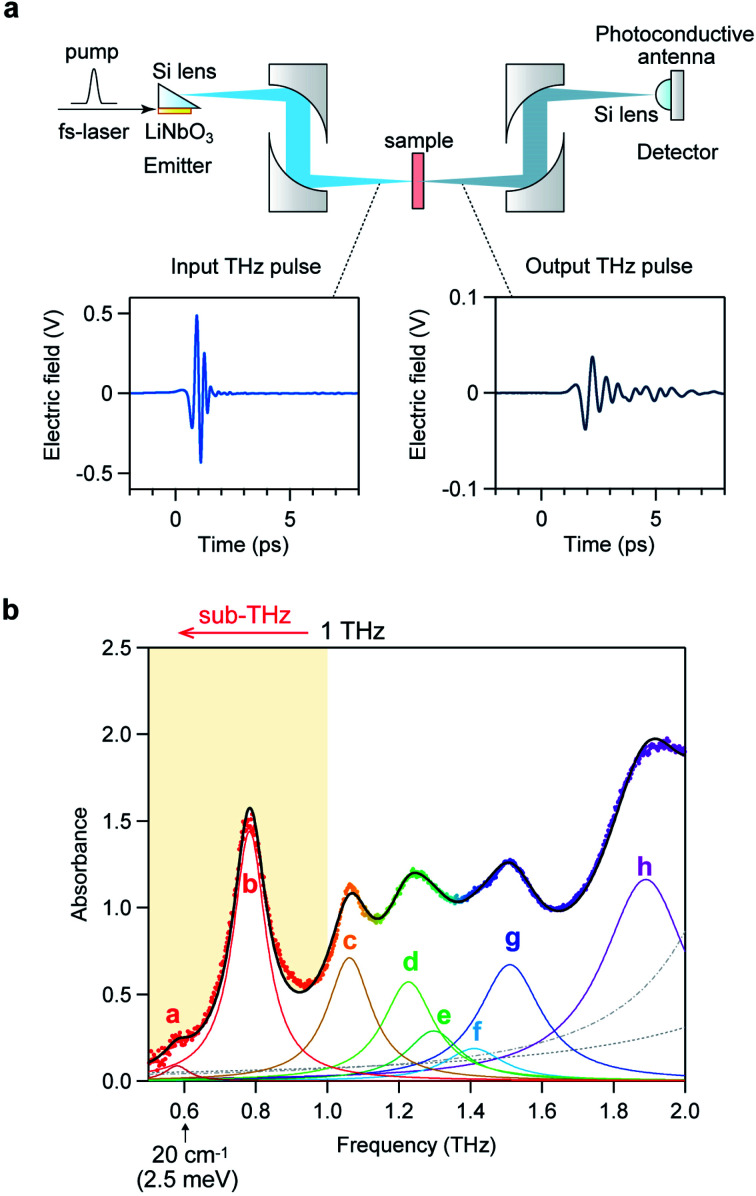
THz-TDS measurement of **RbCoW**. (a) Setup of the THz-TDS measurement system and the temporal waveforms of the input and output THz pulses. (b) THz absorption spectrum of **RbCoW** with peaks at 0.58 (peak a), 0.78 (peak b), 1.06 (peak c), 1.22 (peak d), 1.30 (peak e), 1.41 (peak f), 1.51 (peak g), and 1.89 THz (peak h). Black line shows the fitted curve, coloured lines show the components of each peak, and dots indicate the experimental data. Faint dashed grey lines represent the fitted curves for the higher frequency phonon modes at 2.34 and 2.94 THz.

Optical phonon modes of **RbCoW** were measured by THz-TDS. The optical setup is shown in Fig. 4a. The temporal spectrum of the THz pulse wave was acquired in the transmittance mode. Fourier transformation of the temporal waveform yielded the frequency-dependent power logarithm spectrum. The THz absorption spectrum shows that **RbCoW** has eight absorption peaks: 0.58, 0.78, 1.06, 1.22, 1.30, 1.41, 1.51, and 1.89 THz (Fig. 4b). These observed peaks agree well with peaks a–h in the calculated optical phonon spectrum. Thus, **RbCoW** possesses an extremely low-frequency optical phonon due to the movement of the Rb ion intercalated between the layers. Such low-frequency optical phonons, with only 
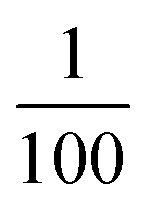
 of the frequency of a CN stretching mode, are rarely observed.

### Temperature-induced switching of low-frequency phonon modes

**Fig. 5:**
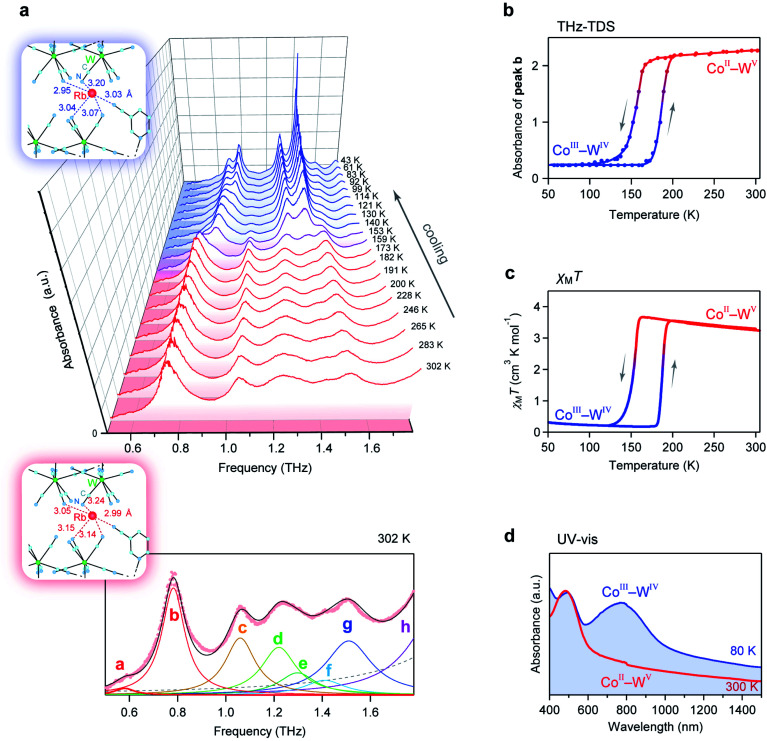
Temperature-induced switching effect of the phonon modes. (a) Temperature dependence of the THz absorption spectra. Upper blue and lower pink frames show the coordination environments around the Rb ion at 130 K and 300 K, respectively. Lower figure shows the fitting of the absorption spectrum at 302 K. Black line shows the fitted curve, coloured lines show the components of each peak, and pink dots indicate the experimental data. Faint dashed grey line represents the fitted curve of the higher frequency phonon mode. (b) Temperature dependence plot of the intensity of peak b at 0.78 THz. (c) *χ*_M_*T vs. T* plot from the magnetic measurement. (d) UV-vis spectra measured at 80 K (blue line) and 300 K (red line).

Next, we investigated the temperature dependence of the optical phonon spectra. The peak positions of peaks a–h are almost constant from 300 K to 150 K. However, the intensities of peaks a–h below 150 K decrease and new peaks appear at 0.87, 1.31, and 1.42 THz (Fig. 5a). During the warming process, the intensities of these new peaks decrease, and peaks a–h are recovered at 190 K (Fig. 5b). The spectral simulation indicates that the optical phonon spectra below 150 K are composed of eight peaks (Fig. S2†): 0.69 (peak i), 0.87 (peak j), 0.91 (peak k), 1.14 (peak l), 1.31 (peak m), 1.42 (peak n), 1.58 (peak o), and 1.75 THz (peak p).

To understand this temperature-induced switching effect, we measured the temperature dependence of the magnetic susceptibility (*χ*_M_) using a superconducting quantum interference device (SQUID) because both Co^II^ and W^V^ have magnetic spins. Fig. 5c shows the product of *χ*_M_ and temperature (*T*) *vs. T* plot. The *χ*_M_*T* value of **RbCoW** at 300 K is 3.24 K cm^3^ mol^−1^. This agrees well with the calculated value of 3.30 K cm^3^ mol^−1^ based on Co^II^ high spin (HS) (*S* = 3/2) and W^V^ (*S* = 1/2) with *g*_Co_ of 2.50 and *g*_W_ of 2.00. The *χ*_M_*T* value decreases at 150 K and becomes 0.23 K cm^3^ mol^−1^. This small *χ*_M_*T* value indicates that the low-temperature phase below 150 K has valence states of Co^III^ low spin (LS) (*S* = 0) and W^IV^ (*S* = 0). The transition temperature in the *χ*_M_*T vs. T* plot agrees with that in the temperature-dependent THz-TDS data. By contrast, in the warming process, the Co^III^(LS)–W^IV^ phase returns to the Co^II^(HS)–W^V^ phase at 190 K. Therefore, the temperature-induced switching effect of the low-frequency phonon mode of **RbCoW** can be explained by a charge-transfer-induced spin transition (CTIST).^56–58^ That is, it is a phase transition between the Co^II^–W^V^ phase and the Co^III^–W^IV^ phase. Fig. 5d shows the UV-vis spectra of the Co^II^–W^V^ phase at 300 K and the Co^III^–W^IV^ phase at 80 K.

The crystal structure of the Co^III^–W^IV^ low-temperature phase has a triclinic structure (space group = *P*1̄) with crystallographic parameters of *a* = 7.2253(14) Å, *b* = 13.340(3) Å, *c* = 13.839(3) Å, *α* = 118.671(8)°, *β* = 100.497(7)°, *γ* = 90.100(6)°, and *V* = 1144.6(4) Å^3^ at 130 K (Fig. S3 and Table S5†), which is isostructural with the Co^II^–W^V^ phase at 300 K. The lattice constants of the *a*-, *b*-, and *c*-axes of the Co^III^–W^IV^ low-temperature phase at 130 K are compressed by 4.2%, 3.1%, and 1.7%, respectively, compared with those of the Co^II^–W^V^ phase at 300 K. As the temperature decreases, the Co^II^(*S* = 3/2)–W^V^ phase shows a charge-transfer-induced phase transition to the Co^III^(*S* = 0)–W^IV^ phase and is accompanied by a spin-crossover from Co^III^(HS) to Co^III^(LS). The ionic radius of Co^II^(HS) is 0.89 Å, while that of Co^III^(LS) is 0.69 Å. In fact, the average Co–N bond length shrinks from 2.11(2) Å at 300 K to 1.90(3) Å at 130 K (Table S6†). The distances between the Rb ion and the surrounding nitrogen atoms of the cyanide ligands are shorter compared to those at 300 K. Thus, the frequency shifts of the optical phonon modes from 0.58 (peak a) to 0.69 THz (peak i) and from 0.78 (peak b) to 0.87 THz (peak j) are caused by a stronger Rb trapping force due to the contraction of the Rb–N distances (Table S7†).

### Photo-induced switching of low-frequency phonon mode

**Fig. 6:**
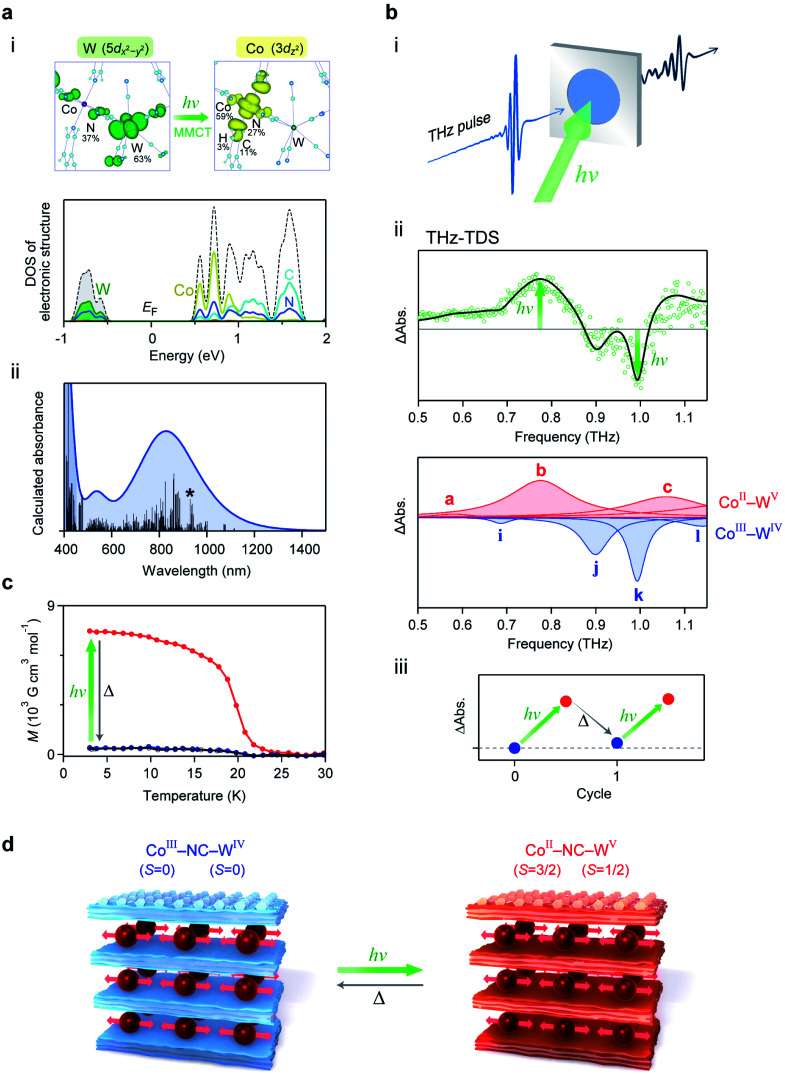
Electronic structure and photo-induced switching of low-frequency phonon mode. (a) (i) Upper figures show the charge density maps of the valence band top (left) and the conduction band bottom (right) of the allowed transition. Lower graph is the density of states (DOS) of the Co^III^–W^IV^ phase near the Fermi energy (*E*_F_) obtained from first-principles electronic structure calculations. Black dotted line shows the total DOS. Dark yellow, green, light blue, and blue lines indicate the partial density of states for Co, W, C, and N, respectively. (ii) Calculated optical absorption spectrum from the transition probabilities of the **RbCoW** Co^III^–W^IV^ phase obtained by first-principles calculation. Black bars show the calculated transition probabilities. Line with an asterisk represents the transition corresponding to the transition between the states of the charge density maps in (i). (b) (i) Illustration of the THz-TDS measurement under photo-irradiation. (ii) Upper shows the differential spectrum between the THz absorption spectra before and after light irradiation (green open circles) and the simulated spectrum (black line). The red and blue peaks in the lower graph represent the components from the Co^II^–W^V^ phase and Co^III^–W^IV^ phase, respectively. Based on the temperature-induced phase transition between the Co^II^–W^V^ and Co^III^–W^IV^ phases in Fig. 5a, the ratio of the peak area between these two phases is 1 : 0.63. Differential spectrum was fitted using the ratio of the peak area between the Co^II^–W^V^ and Co^III^–W^IV^ phases. (iii) Photo-thermal reversibility observed as the difference of the THz absorption spectra at 0.78 THz, where the pre-photoirradiation value is set as the standard. (c) Magnetisation *vs.* temperature plot under a magnetic field of 100 Oe before (blue) and after (red) irradiation with a 785 nm laser at 3 K, and after thermal annealing up to 120 K (black). (d) Illustration of the photo-induced switching between the Co^III^–W^IV^ phase and the Co^II^–W^V^ phase of the 2D layered metal–organic framework.

We investigated the photo-induced switching effect of the low-frequency optical phonon modes of **RbCoW**. At 80 K, **RbCoW** takes a Co^III^–W^IV^ phase and possesses an intense absorption around 780 nm (Fig. 5d). To assign the UV-vis absorption spectrum, the electronic structure of the Co^III^–W^IV^ phase of **RbCoW** was calculated by first-principles calculations using the Vienna *Ab initio* Simulation Package (VASP) program (see Methods). The density of states (DOS) showed that the top of the valence band just below the Fermi energy (*E*_F_) mainly consists of W^IV^ and N components (Co 0%, W 63%, N 37%, C 0%, H 0%), whereas the Co^III^ and N components (Co 59%, W 0%, N 27%, C 11%, H 3%) mainly contribute to the bottom of the conduction band just above *E*_F_ (Fig. 6a(i) and S4†). This indicates that the lowest-energy transition in this compound is a charge transfer from W^IV^ to Co^III^. Fig. 6a(ii) shows the calculated optical absorption spectrum, which well reproduces the experimental spectrum.

Since the Co^III^–W^IV^ phase has an optical transition around 780 nm, 785 nm light was irradiated to the sample inside the THz-TDS system (Fig. 6b(i)). Fig. 6b(ii) shows the observed differential spectrum of the experimental THz spectra before and after irradiation. The absorption peaks at 0.78 THz and 1.08 THz increase by light irradiation, while peaks at 0.90 THz and 0.99 THz decrease. From the simulation based on the change in the ratio between the Co^III^–W^IV^ and Co^II^–W^V^ phases, the increasing components at 0.78 THz and 1.08 THz correspond to peaks b and c of the Co^II^–W^V^ phase, respectively, while the decreasing components at 0.90 THz and 0.99 THz correspond to peaks j and k of the Co^III^–W^IV^ phase, respectively. Additionally, this photo-induced switching effect on the optical phonon mode is repeatedly observed by light irradiation and thermal annealing (5 K → 120 K → 5 K) (Fig. 6b(iii)).

To confirm the mechanism of the photo-induced switching effect, a photoirradiation experiment was performed inside the SQUID magnetometer at 3 K. The observed spontaneous magnetisation with a Curie temperature of 20 K and a coercive field of 4.2 kOe can be explained by the ferromagnetic ordering between the photo-generated Co^II^ and W^V^ (Fig. 6c). Therefore, the photo-induced optical phonon switching of the low-frequency phonon mode originates from the photo-induced phase transition from the Co^III^–W^IV^ to the Co^II^–W^V^ phase, *i.e.*, an optically induced CTIST effect (Fig. 6d).

## Conclusion

From the viewpoint of phonon design, we demonstrate extremely low-frequency optical phonon modes of 0.58 THz (580 GHz, 2.4 meV) and 0.78 THz (780 GHz, 3.2 meV). Such sub-THz optical phonon modes, with only 
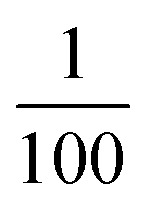
 of the frequency of a CN stretching mode, are successfully realised by sandwiching a heavy monoatomic ion by a 2D metal–organic soft framework. Furthermore, in an isostructural analogue compound where Rb is replaced with Cs, the optical phonon modes are observed at even lower frequencies of 0.57, 0.69, and 0.86 THz because Cs is heavier than Rb (Fig. S5†). This study demonstrates that extremely low-frequency optical phonon modes can be designed by selecting the types of heavy ions to be encapsulated, organic ligands, and transition metal ions. It will be possible to design even lower optical phonon modes by choosing the suitable organic ligands. Sub-THz phonon materials may be useful for electromagnetic wave devices as next-generation wireless communications such as beyond 5G and THz communications in an IoT society.

Furthermore, from the viewpoint of dynamic functions of the low-frequency phonon mode, we show temperature- and photo-induced switching effects on the sub-THz phonon modes. Various functionalities such as magnetism, ionic conduction, and external-stimulation responsivity into the 2D metal–organic framework layers can be incorporated using low-frequency phonon 2D layers to study the synergistic effects between these functionalities and the low-frequency optical phonons. For example, an effective way to switch the phonon frequency by external stimuli is to include external-stimuli responsive units such as spin-crossover,^59–67^ charge-transfer,^56–58,68–76^ and photo-isomerisation units,^77–80^ into the framework. External-stimulation-tuneable THz or sub-THz phonon crystals may lead to the development of devices such as optical filters and optical shutters for THz or sub-THz lights.

## Methods

### Material

Single crystals were prepared by the following procedure. An aqueous solution (2 cm^3^) of cobalt dichloride hexahydrate (0.3 mmol), 3-cyanopyridine (0.6 mmol), and rubidium chloride (2.7 mmol) was placed on one side of the H-shaped tube, while an aqueous solution (2 cm^3^) of rubidium octacyanidetungstate hydrate (0.3 mmol) and rubidium chloride (2.7 mmol) was added on the other side. Additionally, a rubidium chloride aqueous solution (0.35 mol dm^−3^) was used as a buffer solution. After standing for one month, red platelet crystals appeared. The collected crystals were dried under a vacuum or Ar atmosphere at room temperature to give crystals of the target material. Elemental analysis calcd: Co 7.9, W 24.7, Rb 11.5, C 32.3, H 1.1, N 22.6; found: Co 8.1, W 24.7, Rb 11.2, C 32.2, H 1.2, N 22.7.

### Single-crystal X-ray diffraction measurements

Single-crystal X-ray diffraction measurements for **RbCoW** at 300 K and 130 K were performed under a nitrogen flow using a Rigaku R-AXIS Rapid imaging plate area detector with graphite monochromated Mo Kα radiation. The crystal was covered with Apiezon N to prevent hydration. The average correction of the diffraction data was performed in the crystal structure program in Rigaku. The crystal structures at 300 K and 130 K were solved by a direct method using SHELXS-97 on Olex 2-1.2 software. CCDC-1945288 and CCDC-1945289† contain the structural information for the Co^II^–W^V^ phase at 300 K and the Co^III^–W^IV^ phase at 130 K, respectively.

### Phonon mode calculation

First-principles phonon mode calculations were conducted using the Phonon code.^81^ The atomic positions were optimised with an energy cutoff of 500 eV and a 3 × 3 × 3 *k*-mesh until satisfying a 10^−5^ eV pm^−1^ force tolerance in each atom. The phonon mode calculations used 2 × 1 × 1 supercells of the optimised crystal structures. The direct method implemented in the phonon code had a displacement of 2 pm. The transition probabilities of the infrared active optical phonon modes were also calculated. Table S2† shows the list of optical phonon modes.

### THz wave absorption measurements

The absorption properties of the polycrystalline sample in the measurement range of 0.5–2 THz were measured by THz-TDS system of Advantest TAS7400TS in the transmittance mode. The measurement at 293 K was performed by forming a polycrystalline sample into a pellet with a thickness of 0.72 mm. The variable-temperature THz absorption measurement was performed using a pellet sample with a thickness of 1.36 mm by placing it between circular polyethylene plates and pressing with a copper wire to improve the thermal connectivity between the sample and cryostat. The photoirradiation measurement of THz spectroscopy was performed by dispersing **RbCoW** with nujol under an Ar atmosphere and covering between polypropylene plates. The dispersed sample thickness was 0.18 mm.

### Magnetic measurements

The magnetic properties were measured using a Quantum Design MPMS 5 SQUID magnetometer. The sample for magnetic property measurements was prepared by placing a polycrystalline sample in a quartz cell. The magnetic susceptibility of **RbCoW** was measured under 5000 Oe. To measure photo-induced magnetisation, the powder-form sample of **RbCoW** was spread onto tape and placed on the edge of an optical fibre. Then 785 nm continuous wave laser light was irradiated at 3 K.

### Electronic structure calculation

Periodic electronic structure calculation of the Co^II^–W^V^ phase was carried out using the VASP program.^82,83^ To take into account the van der Waals interaction, the calculations were based on the van der Waals density functional rev-vdW-DF2. The *U*–*J* parameters were set to 7.0 eV for Co 3d electrons and 2.6 eV for W 5d electrons. The wave functions were based on the plane wave with an energy cutoff of 500 eV. A *k*-mesh of 3 × 3 × 3 was used for Brillouin zone samplings. The electronic iteration convergence was set to 1 × 10^−9^ eV. Partial DOS were calculated by integrating over the volumes of atomic spheres on atoms with the following radii: W: 1.30 Å, Co: 1.16 Å, N: 0.75 Å, C: 0.77 Å, Rb: 2.16 Å and H: 0.32 Å. The distributions of the charge densities were estimated by Bader analysis.

## Conflicts of interest

There are no conflicts to declare.

## Supplementary Material

SC-011-D0SC02605K-s001

SC-011-D0SC02605K-s002

SC-011-D0SC02605K-s003

SC-011-D0SC02605K-s004
