# Supplementary material for: Extremely low-frequency phonon material and its temperature- and photo-induced switching effects
Source: Chem Sci. 2020 Aug 17;11(33):8989–98. doi: 10.1039/d0sc02605k (PMC8163449; doi:10.1039/d0sc02605k)
Supplement: SC-011-D0SC02605K-s001 [file SC-011-D0SC02605K-s001.pdf]

Electronic Supplementary Material (ESI) for Chemical Science.
This journal is © The Royal Society of Chemistry 2020

Extremely low-frequency phonon material and its temperature- and photo-induced switching effects

Takaya Yoshida,[1] Koji Nakabayashi,[1] Hiroko Tokoro,[1,2] Marie Yoshikiyo,[1]
Asuka Namai,[1] Kenta Imoto,[1] Kouji Chiba,[3] and Shin-ichi Ohkoshi[*,1]

[1] *Department of Chemistry, School of Science, The University of Tokyo*
7-3-1 Hongo, Bunkyo-ku, Tokyo 113-0033, Japan.
[2] *Division of Materials Science, Faculty of Pure and Applied Sciences, University of Tsukuba*
1-1-1 Tennodai, Tsukuba, Ibaraki 305-8573, Japan.
[3] *Material Science Div., MOLSIS Inc., Tokyo Daia Bldg.*
1-28-38 Shinkawa, Chuo-ku, Tokyo 104-0033, Japan.

*To whom correspondence should be addressed
E-mail: ohkoshi@chem.s.u-tokyo.ac.jp

Table of Contents: **Page**

1	Legend for Supporting Movies	S2
2	Experimental details	S3
3	Crystal structure analysis of Co^{II}–W^{V} phase of **RbCoW** (Table S1)	S4
4	First-principles phonon mode calculation (Table S2-S4, Figure S1)	S5–S8
5	Temperature-induced switching effect on the low-frequency phonon mode (Figure S2)	S9
6	Crystal structure analysis of the Co^{III}–W^{IV} phase of **RbCoW** (Figure S3, Table S5)	S10,S11
7	Comparison of the crystal structure of **RbCoW** between the Co^{II}–W^{V} phase and Co^{III}–W^{IV} phase (Tables S6, S7)	S12
8	First-principles electronic structure calculation of the Co^{III}–W^{IV} phase (Figure S4)	S13
9	THz absorption spectrum of **CsCoW** (Figure S5)	S14

Section 1. Legend for Supporting Movies

Movie S1. "Low-frequency phonon millefeuille" based on metal–organic framework. Initial part of the movie shows monoatomic molecules shown with red balls moving freely in the gas phase or liquid phase (translational mode). Next, a monoatomic molecule is caught by a two-dimensional (2D) metal–organic soft framework (similar to pastry sheets), and the translational mode switches to a vibrational mode (optical phonon mode) with a low vibrational frequency. The layers pile up with the 2D layers softly sandwiching the low-frequency optical phonon due to heavy atoms. Here we call it "low-frequency phonon millefeuille".

Movie S2. Calculated phonon modes of RbCoW. The first part of the movie shows the list of low-frequency optical phonon modes. Then, the movie shows the atomic movements of the phonon modes corresponding to **peaks a** (0.883 THz), **b** (0.997 THz), **c** (1.136 THz), **d** (1.453 THz), **e** (1.682 THz), **f** (1.805 THz), **g** (1.923 THz), and **h** (2.102 THz) in the optical phonon spectrum. **Peaks a**, **b** and **c** are assigned to the phonon modes of Rb ion vibrations in the *ab* plane. Atomic movement of **peak d** is due to Rb ion oscillation along the crystallographic *c*-axis. In these phonon modes, the 3-cyanopyridine and cyanide ligands sway together with the Rb ions, supporting the Rb movements. **Peaks e** and **f** are attributed to the combination of transverse translational modes of W–CN–Co and 3-cyanopyridine ligand rotation, and **peaks g** and **h** originate from the transverse librational modes of W–CN–Co and 3-cyanopyridine ligand rotation. Red, blue, green, ocher, and grey balls represent Rb, Co, W, C, and N atoms, respectively.

Section 2. Experimental details

Synthesis. Polycrystalline sample of **RbCoW** was synthesised by reacting a 15 mL aqueous solution of cobalt (II) dichloride (2.3 mmol), 3-cyanopyridine (3.0 mmol), rubidium chloride (7.6 mmol) with a 15 mL solution of rubidium octacyanidetungstate hydrate (1.5 mmol) and rubidium chloride (0.45 mol dm^{-3}). Stirring for 24 hours and subsequently standing under vacuum conditions at room temperature yielded a red-brown powder.

Measurements. The chemical formulas of the metal assemblies were estimated by micro-analytical methods for carbon, nitrogen, and hydrogen. The chemical composition of the metal ions (Rb, Co, W) were estimated by inductively coupled plasma mass spectroscopy using Agilent 7700x. Infrared absorption spectrum of **RbCoW** was obtained by JASCO FT/IR-4100 and Shimadzu FTIR-8200 spectrometers. The sample was prepared by standing under an Ar atmosphere, dispersing with nujol, and sandwiching between CaF_2 plates. UV-visible absorption spectra of **RbCoW** were obtained by Shimadzu 3600 Plus. The sample was prepared by standing under an Ar atmosphere, dispersing with nujol, and sandwiching between polypropylene plates. The temperature-dependent spectra of UV-vis absorption were obtained using Oxford MicrostatHe2. Additionally, 785 nm CW laser (48.5 mW) made by Applied Techno was used for photo-irradiation.

Section 3. Crystal structure analysis of Co^{II}–W^{V} phase of RbCoW

Table S1. Crystallographic data and structure refinement of the Co^{II}–W^{V} phase of **RbCoW** at 300 K.

Compound	**RbCoW** (Co^{II}–W^{V} phase)
Empirical formula	$C_{20}H_{8}CoN_{12}RbW$
Formula weight	744.63
Temperature/K	300(2)
Crystal system	triclinic
Space group	$P\bar{1}$
a/Å	7.5298(4)
b/Å	13.7545(7)
c/Å	14.0687(6)
α/°	119.276(4)
β/°	101.020(6)
γ/°	89.974(6)
V/Å^3	1240.15(12)
Z	2
ρ_{calc} /g cm^{-3}	1.994
μ/mm^{-1}	7.286
F(000)	700.0
Crystal size/mm^3	0.204 × 0.042 × 0.024
Radiation	MoKα (λ = 0.71073)
2θ range for data collection/°	6.186 to 54.934
Index ranges	$-9 \leq h \leq 9$<br>$-17 \leq k \leq 17$<br>$-18 \leq l \leq 18$
Reflections collected	11945
Independent reflections	5621 [R_{int} = 0.0911, R_{sigma} = 0.1318]
Data/restraints/parameters	5621/23/256
Goodness-of-fit on F^2	1.140
Final R indexes [I>=2σ (I)]	R_1 = 0.0829<br>wR_2 = 0.1260
Final R indexes [all data]	R_1 = 0.1152<br>wR_2 = 0.1352
Largest diff. peak/hole / e Å^{-3}	2.10/−4.75

Section 4. First-principles phonon mode calculation

Table S2-1. Calculated optical phonon modes obtained by first-principles phonon mode calculations of **RbCoW**.

Frequency [THz]	Wavenumber [cm^{-1}]	Irreducible representation	Infrared intensity [×10^{-4}]
0.883	29.4	A_u	3.4880
0.997	33.2	A_u	13.9400
1.136	37.9	A_u	17.6050
1.166	38.9	A_g	0.0000
1.286	42.9	A_g	0.0000
1.453	48.4	A_u	8.2990
1.490	49.7	A_g	0.0000
1.571	52.4	A_g	0.0000
1.603	53.4	A_g	0.0000
1.682	56.1	A_u	10.0230
1.745	58.2	A_g	0.0000
1.805	60.2	A_u	4.6260
1.912	63.7	A_g	0.0000
1.923	64.1	A_u	3.2580
1.998	66.6	A_g	0.0000
2.102	70.1	A_u	30.1720
2.172	72.4	A_g	0.0000
2.188	72.9	A_u	0.1350
2.244	74.8	A_g	0.0000
2.341	78.0	A_g	0.0000
2.408	80.3	A_g	0.0000
2.484	82.8	A_u	2.8260
2.546	84.9	A_g	0.0000
2.569	85.6	A_u	6.1700
2.607	86.9	A_g	0.0000
2.657	88.6	A_u	17.8610
2.893	96.4	A_g	0.0000
2.962	98.7	A_u	0.8400
3.042	101.4	A_g	0.0000
3.047	101.6	A_u	4.2980
3.173	105.8	A_g	0.0000
3.284	109.5	A_u	0.0010
3.354	111.8	A_g	0.0000
3.367	112.2	A_u	1.7940
3.466	115.5	A_u	5.1640
3.481	116.0	A_u	2.4870
3.508	116.9	A_g	0.0000
3.566	118.9	A_g	0.0000
3.751	125.0	A_g	0.0000
3.948	131.6	A_u	0.1650
4.014	133.8	A_g	0.0000
4.097	136.6	A_u	1.9980

Frequency [THz]	Wavenumber [cm^{-1}]	Irreducible representation	Infrared intensity [×10^{-4}]
4.182	139.4	A_u	2.5720
4.236	141.2	A_g	0.0000
4.374	145.8	A_g	0.0000
4.521	150.7	A_u	56.5530
4.523	150.8	A_g	0.0000
4.601	153.4	A_u	44.8030
4.708	156.9	A_g	0.0000
4.755	158.5	A_g	0.0000
4.830	161.0	A_u	71.1950
5.000	166.7	A_u	75.3830
5.081	169.4	A_g	0.0000
5.107	170.2	A_g	0.0000
5.113	170.4	A_u	354.8910
5.278	175.9	A_u	70.9100
5.418	180.6	A_u	33.8680
5.576	185.9	A_u	8.4450
5.602	186.7	A_u	30.5130
5.693	189.8	A_g	0.0000
5.788	192.9	A_u	0.9050
5.832	194.4	A_g	0.0000
5.879	196.0	A_u	0.6440
5.985	199.5	A_g	0.0000
6.042	201.4	A_g	0.0000
6.203	206.8	A_u	3.4400
6.246	208.2	A_u	2.8060
6.296	209.9	A_u	3.4540
6.379	212.6	A_g	0.0000
6.533	217.8	A_g	0.0000
6.563	218.8	A_u	0.2040
6.613	220.4	A_g	0.0000
6.617	220.6	A_u	0.4300
6.733	224.4	A_g	0.0000
7.006	233.5	A_u	176.0970
7.236	241.2	A_g	0.0000
7.307	243.6	A_u	57.2250
7.502	250.1	A_u	29.7720
7.623	254.1	A_u	0.2270
9.368	312.3	A_g	0.0000
9.485	316.2	A_u	2.1460
9.684	322.8	A_g	0.0000
9.745	324.8	A_g	0.0000
9.832	327.7	A_u	0.3220

Frequency [THz]	Wavenumber [cm^{-1}]	Irreducible representation	Infrared intensity [×10^{-4}]
9.844	328.1	A_g	0.0000
9.994	333.1	A_u	7.5450
10.449	348.3	A_u	125.1880
10.492	349.7	A_g	0.0000
10.661	355.4	A_u	35.5870
10.681	356.0	A_g	0.0000
10.716	357.2	A_g	0.0000
10.759	358.6	A_u	4.5580
10.762	358.7	A_g	0.0000
10.768	358.9	A_u	2.6290
10.793	359.8	A_u	0.5130
10.950	365.0	A_g	0.0000
10.975	365.8	A_u	18.5100
11.129	371.0	A_u	30.4830
11.153	371.8	A_g	0.0000
11.313	377.1	A_g	0.0000
11.430	381.0	A_u	0.6020
11.692	389.7	A_g	0.0000
11.734	391.1	A_u	8.6860
11.748	391.6	A_g	0.0000
11.769	392.3	A_u	48.5640
11.988	399.6	A_g	0.0000
12.017	400.6	A_u	48.7900
12.148	404.9	A_g	0.0000
12.230	407.7	A_u	96.5540
12.287	409.6	A_g	0.0000
12.315	410.5	A_u	10.4700
12.387	412.9	A_g	0.0000
12.391	413.0	A_u	3.2700
12.586	419.5	A_u	3.1320
12.593	419.8	A_g	0.0000
12.780	426.0	A_g	0.0000
12.788	426.3	A_u	20.2580
12.890	429.7	A_g	0.0000
12.964	432.1	A_u	31.6900
12.977	432.6	A_g	0.0000
13.156	438.5	A_u	0.9030
13.191	439.7	A_g	0.0000
13.303	443.4	A_u	53.7610
13.359	445.3	A_g	0.0000
13.463	448.8	A_u	1.6350
13.493	449.8	A_g	0.0000

Table S2-2. Calculated optical phonon modes obtained by first-principles phonon mode calculations of **RbCoW**.

Frequency [THz]	Wavenumber [cm^{-1}]	Irreducible representation	Infrared intensity [×10^{-4}]
13.667	455.6	A_u	2.5030
13.759	458.6	A_g	0.0000
13.824	460.8	A_g	64.9270
13.824	460.8	A_u	0.0010
13.951	465.0	A_u	83.4780
14.036	467.9	A_g	0.0000
14.113	470.4	A_u	11.5030
14.126	470.9	A_g	0.0000
14.157	471.9	A_u	3.7790
14.201	473.4	A_g	0.0000
14.349	478.3	A_u	0.2710
14.528	484.3	A_u	34.8430
14.768	492.3	A_g	0.0000
16.520	550.7	A_u	20.3140
16.524	550.8	A_g	0.0000
16.651	555.0	A_u	0.4780
16.684	556.1	A_g	0.0000
16.717	557.2	A_u	20.0610
16.728	557.6	A_g	0.0000
16.816	560.5	A_g	0.0000
16.859	562.0	A_u	17.7180
19.264	642.1	A_g	0.0000
19.281	642.7	A_u	6.3830
19.287	642.9	A_g	0.0000
19.350	645.0	A_u	72.4030
20.552	685.1	A_g	0.0000
20.564	685.5	A_u	351.0870
20.628	687.6	A_g	0.0000
20.722	690.7	A_u	178.4790
23.684	789.5	A_g	0.0000
23.707	790.2	A_u	1.6890
23.723	790.8	A_g	0.0000
23.730	791.0	A_u	2.8170
24.002	800.1	A_u	118.9470
24.075	802.5	A_g	0.0000
24.429	814.3	A_g	0.0000
24.545	818.2	A_u	133.3730
27.084	902.8	A_u	71.2950
27.094	903.1	A_g	0.0000
27.129	904.3	A_g	0.0000
27.149	905.0	A_u	23.1170
28.238	941.3	A_u	3.2230
28.239	941.3	A_g	0.0000
28.479	949.3	A_g	0.0000
28.528	950.9	A_u	3.2320
29.536	984.5	A_g	0.0000
29.564	985.5	A_u	0.4940
30.051	1001.7	A_u	0.7200
30.123	1004.1	A_g	0.0000
30.454	1015.1	A_g	0.0000
30.486	1016.2	A_u	0.1820
30.641	1021.4	A_g	0.0000
30.643	1021.4	A_u	0.1070
31.416	1047.2	A_g	0.0000
31.432	1047.7	A_u	18.3140
31.446	1048.2	A_g	0.0000
31.471	1049.0	A_u	79.9600
33.390	1113.0	A_u	4.8130
33.411	1113.7	A_g	0.0000
33.494	1116.5	A_u	2.2470
33.575	1119.2	A_g	0.0000
35.731	1191.0	A_u	59.6980
35.732	1191.1	A_g	0.0000
35.753	1191.8	A_u	11.4290
35.852	1195.1	A_g	0.0000
36.097	1203.2	A_u	2.5860
36.103	1203.4	A_g	0.0000
36.161	1205.4	A_g	0.0000
36.185	1206.2	A_u	0.4180
38.585	1286.2	A_g	0.0000
38.610	1287.0	A_u	0.4450
38.692	1289.7	A_g	0.0000
38.716	1290.5	A_u	0.1360
39.531	1317.7	A_u	0.0220
39.541	1318.0	A_g	0.0000
39.610	1320.3	A_g	0.0000
39.626	1320.9	A_u	0.4020
42.077	1402.6	A_g	0.0000
42.095	1403.2	A_u	7.5080
42.098	1403.3	A_g	0.0000
42.102	1403.4	A_u	3.5130
43.811	1460.4	A_g	0.0000
43.813	1460.4	A_u	0.0010
43.869	1462.3	A_g	0.0000
43.874	1462.5	A_u	0.0040
46.568	1552.3	A_g	0.0000
46.575	1552.5	A_u	21.9990
46.593	1553.1	A_g	0.0000
46.614	1553.8	A_u	3.3100
47.397	1579.9	A_g	0.0000
47.400	1580.0	A_u	60.6630
47.419	1580.6	A_u	10.6150
47.450	1581.7	A_g	0.0000
63.791	2126.4	A_u	583.2660
63.875	2129.2	A_g	0.0000
64.065	2135.5	A_u	85.2680
64.093	2136.4	A_g	0.0000
64.153	2138.4	A_g	0.0000
64.215	2140.5	A_u	35.2770
64.516	2150.5	A_u	1671.552
64.655	2155.2	A_g	0.0000
64.699	2156.6	A_u	332.9170
64.739	2158.0	A_g	0.0000
64.809	2160.3	A_u	152.2790
64.887	2162.9	A_g	0.0000
64.973	2165.8	A_u	92.6680
65.150	2171.7	A_g	0.0000
65.232	2174.4	A_u	7.5780
65.236	2174.5	A_g	0.0000
68.061	2268.7	A_g	0.0000
68.067	2268.9	A_u	5.4740
68.207	2273.6	A_u	0.1530
68.231	2274.4	A_g	0.0000
92.449	3081.6	A_g	0.0000
92.508	3083.6	A_u	6.2680
92.520	3084.0	A_g	0.0000
92.520	3084.0	A_u	4.1160
92.701	3090.0	A_u	33.0030
92.739	3091.3	A_g	0.0000
92.841	3094.7	A_g	0.0000
92.845	3094.8	A_u	5.1740
92.994	3099.8	A_u	1.1500
92.997	3099.9	A_g	0.0000
93.055	3101.8	A_g	0.0000
93.061	3102.0	A_u	9.5130
93.286	3109.5	A_u	1.9800
93.289	3109.6	A_g	0.0000
93.321	3110.7	A_g	0.0000
93.329	3111.0	A_u	7.7770

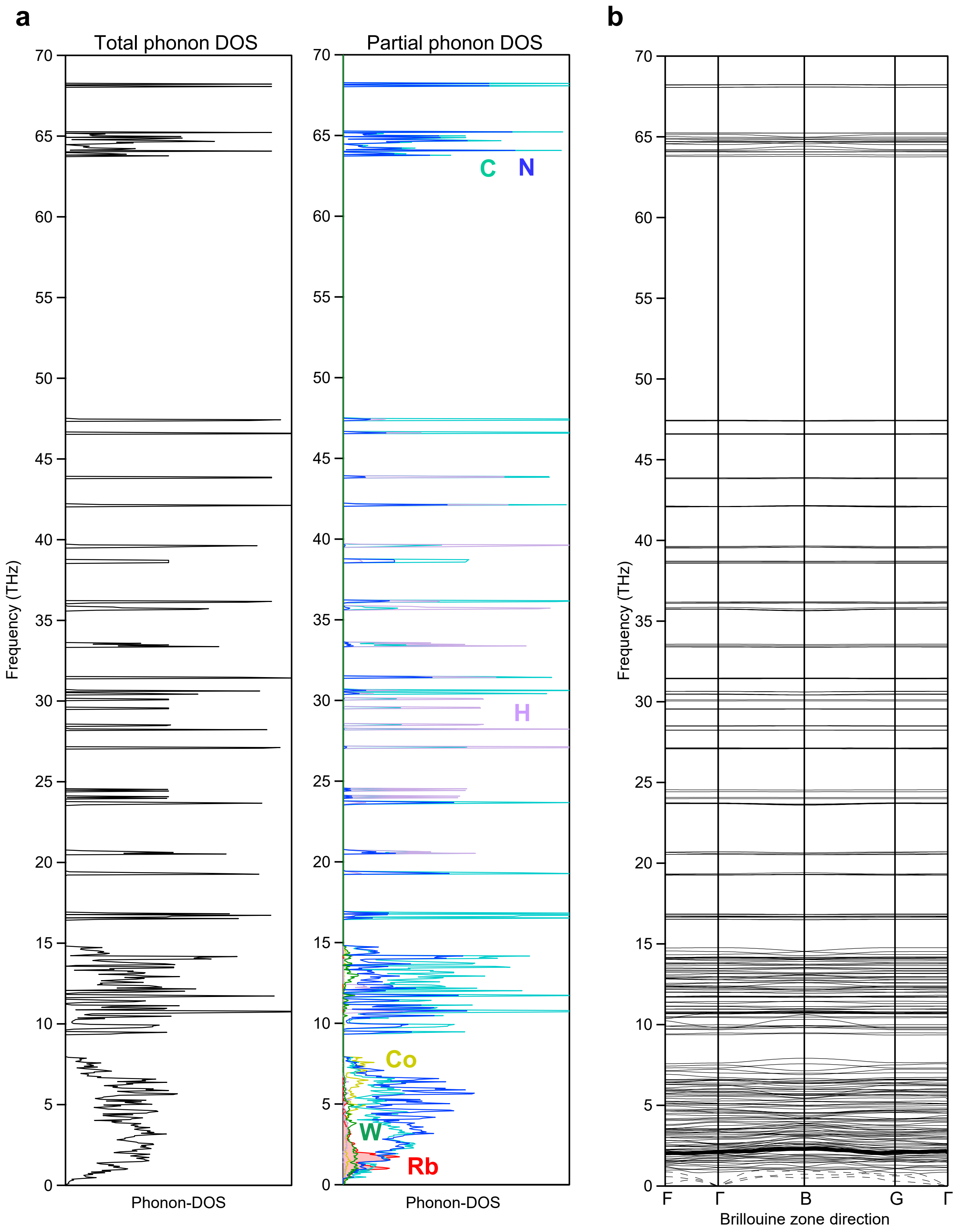

Figure S1. (a) Total phonon-DOS of the **RbCoW** Co^{II}–W^{V} phase (black line) and partial phonon-DOS of Rb (red line), Co (dark yellow line), W (green line), C (light blue line), N (blue line), and H (purple line). (b) Phonon dispersion of the **RbCoW** Co^{II}–W^{V} phase.

Table S3. Cell parameters of **RbCoW** before and after structural optimization, and their differences (Δ). Initial cell parameters before structural optimization are determined by single crystal structural analysis. Optimized cell parameters are used for the phonon mode calculations.

	Single crystal structural analysis	Optimized structure	Δ (%)
a / Å	15.060	15.026	−0.23
b / Å	13.755	13.728	−0.20
c / Å	14.069	14.036	−0.24
α / °	119.28	119.25	−0.03
β / °	101.02	100.97	−0.05
γ / °	89.97	90.00	0.03
V / Å^3	2480.3	2465.14	−0.61

Table S4. Atomic positions of **RbCoW** after structural optimization.

Atom label	x	y	z
W	0.77481	0.82203	0.13874
W	0.27481	0.82203	0.13874
W	0.72519	0.17797	0.86126
W	0.22519	0.17797	0.86126
Rb	0.62245	0.06635	0.45143
Rb	0.12245	0.06635	0.45143
Rb	0.87755	0.93365	0.54857
Rb	0.37755	0.93365	0.54857
Co	0	0.5	0
Co	0.5	0.5	0
Co	0	0	0
Co	0.5	0	0
N	0.60417	0.62165	0.04534
N	0.10417	0.62165	0.04534
N	0.89583	0.37835	0.95466
N	0.39583	0.37835	0.95466
N	0.60168	0.93117	0.05601
N	0.10168	0.93117	0.05601
N	0.89832	0.06883	0.94399
N	0.39832	0.06883	0.94399
N	0.90872	0.61634	0.05768
N	0.40872	0.61634	0.05768
N	0.59128	0.38366	0.94232
N	0.09128	0.38366	0.94232
N	0.91108	0.94325	0.05887
N	0.41108	0.94325	0.05887
N	0.58892	0.05675	0.94113
N	0.08892	0.05675	0.94113
N	0.72952	0.67669	0.85972
N	0.22952	0.67669	0.85972
N	0.77048	0.32331	0.14028
N	0.27048	0.32331	0.14028
N	0.95812	0.89536	0.34534
N	0.45812	0.89536	0.34534
N	0.54188	0.10464	0.65466
N	0.04188	0.10464	0.65466
N	0.70362	0.84949	0.35927
N	0.20362	0.84949	0.35927
N	0.79638	0.15051	0.64073
N	0.29638	0.15051	0.64073
N	0.77934	0.09525	0.32148
N	0.27934	0.09525	0.32148
N	0.72066	0.90475	0.67852
N	0.22066	0.90475	0.67852
N	0.53316	0.15481	0.15742
N	0.03316	0.15481	0.15742
N	0.96684	0.84519	0.84258
N	0.46684	0.84519	0.84258
N	0.80153	0.40764	0.40998
N	0.30153	0.40764	0.40998
N	0.69847	0.59236	0.59002
N	0.19847	0.59236	0.59002
N	0.53959	0.51809	0.16461
N	0.03959	0.51809	0.16461
N	0.96041	0.48191	0.83539
N	0.46041	0.48191	0.83539
N	0.81851	0.68573	0.4425
N	0.31851	0.68573	0.4425
N	0.68149	0.31427	0.5575
N	0.18149	0.31427	0.5575
C	0.66636	0.68975	0.07917
C	0.16636	0.68975	0.07917
C	0.83364	0.31025	0.92083
C	0.33364	0.31025	0.92083
C	0.66098	0.89122	0.08701
C	0.16098	0.89122	0.08701
C	0.83902	0.10878	0.91299
C	0.33902	0.10878	0.91299
C	0.85906	0.68566	0.08983
C	0.35906	0.68566	0.08983
C	0.64094	0.31434	0.91017
C	0.14094	0.31434	0.91017
C	0.86372	0.89968	0.08744
C	0.36372	0.89968	0.08744
C	0.63628	0.10032	0.91256
C	0.13628	0.10032	0.91256
C	0.74578	0.72962	0.95755
C	0.24578	0.72962	0.95755
C	0.75422	0.27038	0.04245
C	0.25422	0.27038	0.04245
C	0.89538	0.86757	0.27034
C	0.39538	0.86757	0.27034
C	0.60462	0.13243	0.72966
C	0.10462	0.13243	0.72966
C	0.72626	0.83356	0.27772
C	0.22626	0.83356	0.27772
C	0.77374	0.16644	0.72228
C	0.27374	0.16644	0.72228
C	0.78007	0.0001	0.25702
C	0.28007	0.0001	0.25702
C	0.71993	0.9999	0.74298
C	0.21993	0.9999	0.74298
C	0.96433	0.20487	0.20596
C	0.46433	0.20487	0.20596
C	0.53567	0.79513	0.79404
C	0.03567	0.79513	0.79404
C	0.97872	0.30578	0.30673
C	0.47872	0.30578	0.30673
C	0.52128	0.69422	0.69327
C	0.02128	0.69422	0.69327
C	0.56734	0.35684	0.3597
C	0.06734	0.35684	0.3597
C	0.93266	0.64316	0.6403
C	0.43266	0.64316	0.6403
C	0.63888	0.30549	0.30816
C	0.13888	0.30549	0.30816
C	0.86112	0.69451	0.69184
C	0.36112	0.69451	0.69184
C	0.61903	0.20371	0.20647
C	0.11903	0.20371	0.20647
C	0.88097	0.79629	0.79353
C	0.38097	0.79629	0.79353
C	0.72971	0.35847	0.36112
C	0.22971	0.35847	0.36112
C	0.77029	0.64153	0.63888
C	0.27029	0.64153	0.63888
C	0.97428	0.53236	0.22279
C	0.47428	0.53236	0.22279
C	0.52572	0.46764	0.77721
C	0.02572	0.46764	0.77721
C	0.9933	0.58665	0.33919
C	0.4933	0.58665	0.33919
C	0.5067	0.41335	0.66081
C	0.0067	0.41335	0.66081
C	0.58257	0.62891	0.39815
C	0.08257	0.62891	0.39815
C	0.91743	0.37109	0.60185
C	0.41743	0.37109	0.60185
C	0.65111	0.60754	0.33714
C	0.15111	0.60754	0.33714
C	0.84889	0.39246	0.66286
C	0.34889	0.39246	0.66286
C	0.62687	0.5509	0.21995
C	0.12687	0.5509	0.21995
C	0.87313	0.4491	0.78005
C	0.37313	0.4491	0.78005
C	0.74365	0.64868	0.39394
C	0.24365	0.64868	0.39394
C	0.75635	0.35132	0.60606
C	0.25635	0.35132	0.60606
H	0.89574	0.16216	0.1618
H	0.39574	0.16216	0.1618
H	0.60426	0.83784	0.8382
H	0.10426	0.83784	0.8382
H	0.92046	0.34364	0.34158
H	0.42046	0.34364	0.34158
H	0.57954	0.65636	0.65842
H	0.07954	0.65636	0.65842
H	0.58303	0.43545	0.43954
H	0.08303	0.43545	0.43954
H	0.91697	0.56455	0.56046
H	0.41697	0.56455	0.56046
H	0.67289	0.16123	0.16432
H	0.17289	0.16123	0.16432
H	0.82711	0.83877	0.83568
H	0.32711	0.83877	0.83568
H	0.9046	0.50052	0.17354
H	0.4046	0.50052	0.17354
H	0.5954	0.49948	0.82646
H	0.0954	0.49948	0.82646
H	0.93766	0.59867	0.38235
H	0.43766	0.59867	0.38235
H	0.56234	0.40133	0.61765
H	0.06234	0.40133	0.61765
H	0.60057	0.67687	0.48936
H	0.10057	0.67687	0.48936
H	0.89943	0.32313	0.51064
H	0.39943	0.32313	0.51064
H	0.67828	0.53426	0.1699
H	0.17828	0.53426	0.1699
H	0.82172	0.46574	0.8301
H	0.32172	0.46574	0.8301

Section 5. Temperature-induced switching effect on the low-frequency phonon mode

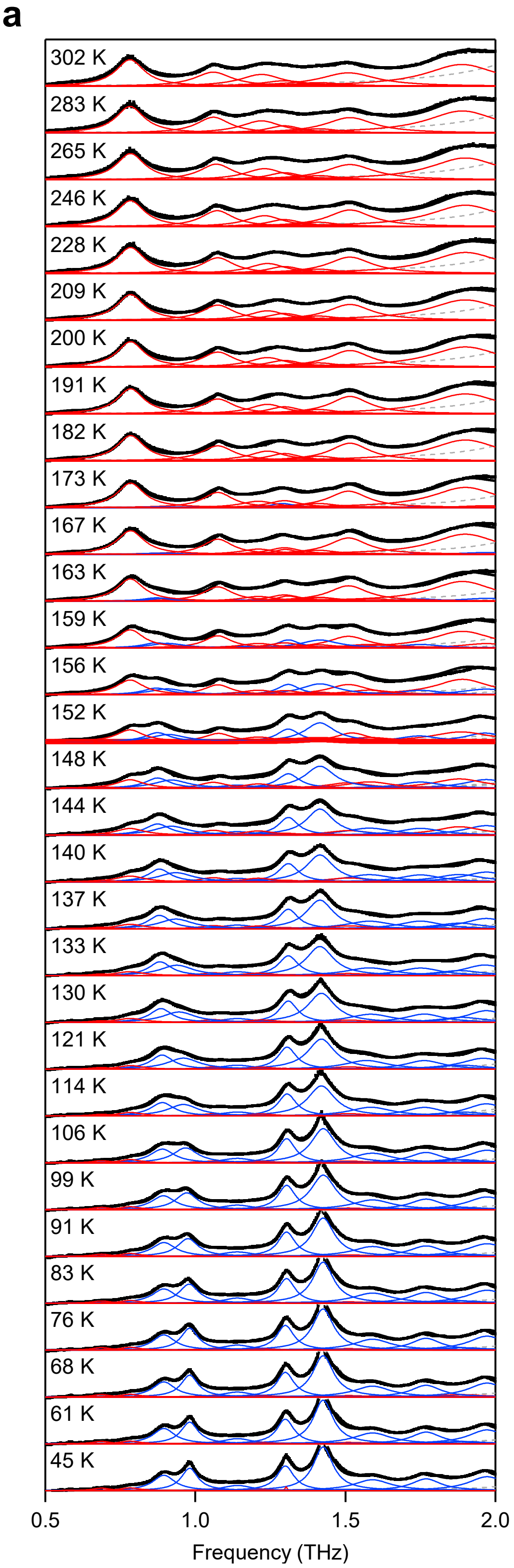

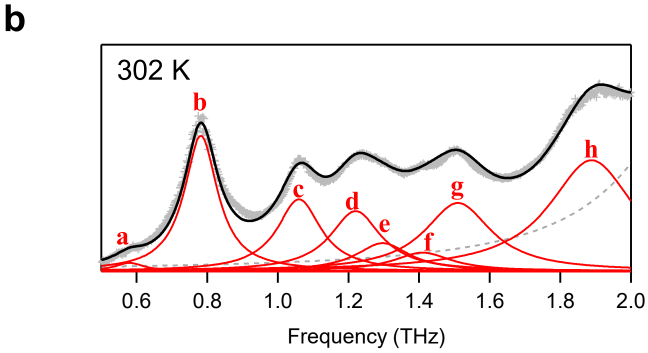

Co^{II}-W^{V} phase	a	b	c	d	e	f	g	h
Position (THz)	0.58	0.78	1.06	1.22	1.30	1.41	1.51	1.89
FWHM (THz)	0.10	0.12	0.15	0.18	0.19	0.20	0.23	0.29
Area	0.02	0.41	0.29	0.28	0.14	0.10	0.41	0.86

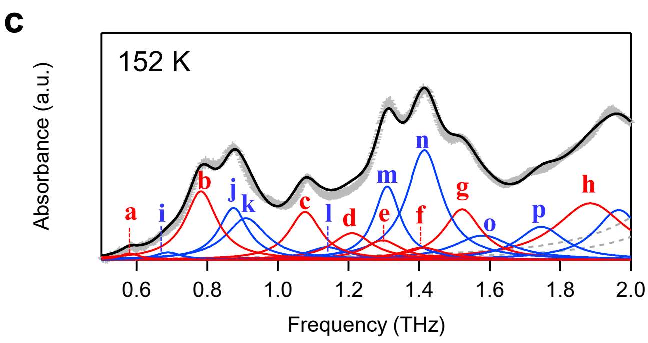

Co^{II}-W^{V} phase	a	b	c	d	e	f	g	h
Position (THz)	0.58	0.78	1.08	1.21	1.30	1.41	1.52	1.89
FWHM (THz)	0.07	0.11	0.11	0.14	0.13	0.15	0.13	0.24
Area	0.01	0.17	0.12	0.09	0.06	0.04	0.15	0.31
**Co^{III}-W^{IV} phase**	**i**	**j**	**k**	**l**	**m**	**n**	**o**	**p**
Position (THz)	0.69	0.87	0.91	1.14	1.31	1.42	1.58	1.75
FWHM (THz)	0.09	0.10	0.14	0.14	0.09	0.12	0.20	0.16
Area	0.01	0.12	0.13	0.04	0.15	0.31	0.11	0.12

Figure S2. (a) Peak analyses of the temperature-dependent THz absorption spectra. THz absorption spectra at (b) 302 K and (c) 152 K. Red lines, blue lines, and black lines indicate the peak components from the Co^{II}–W^{V} phase, those from the Co^{III}–W^{IV} phase, and the total calculated spectra of the Co^{II}–W^{V} and Co^{III}–W^{IV} phases, respectively. Grey dots show the experimental data. Faint dashed grey lines represent the fitted curve of the higher frequency phonon modes.

Section 6. Crystal structure analysis of the Co^{III}–W^{IV} phase of RbCoW

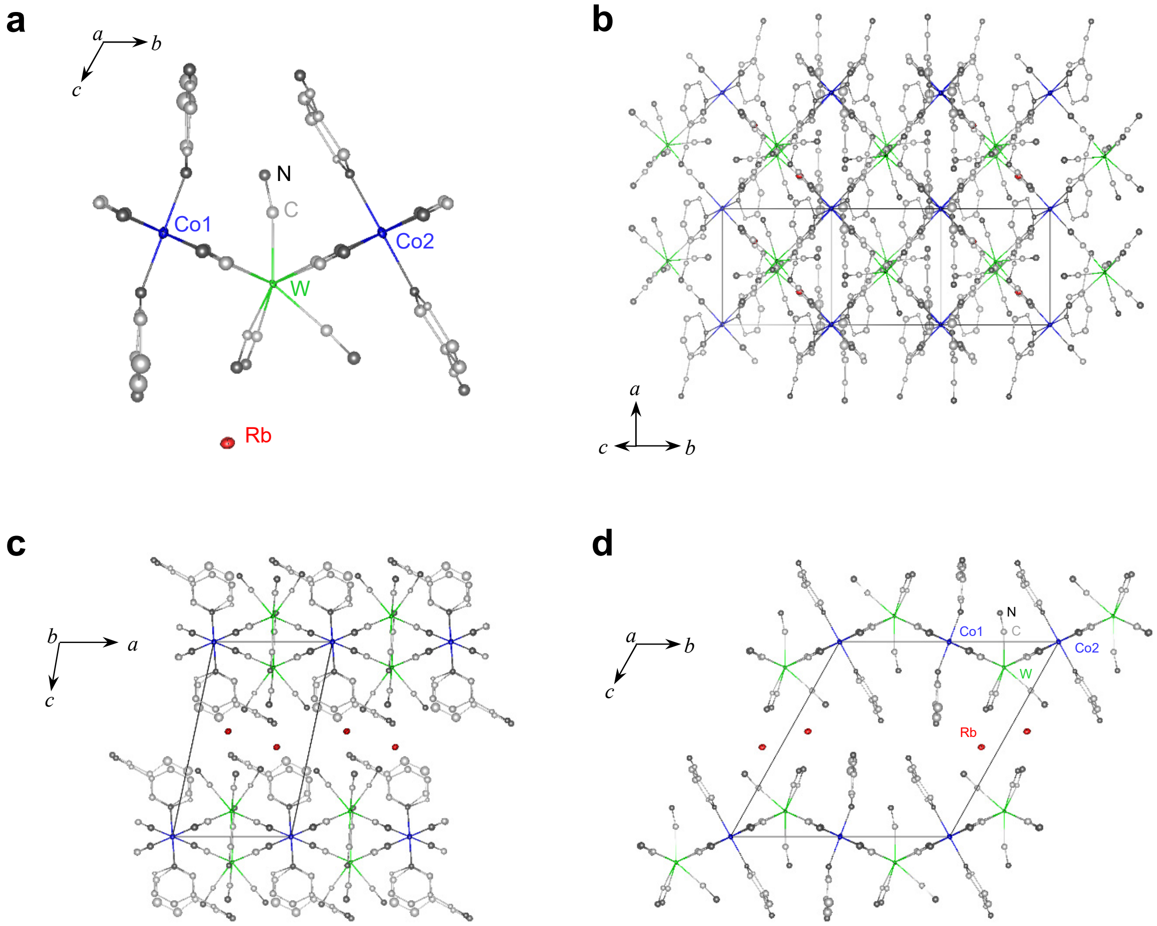

Figure S3. Crystal structure of the Co^{III}–W^{IV} phase of **RbCoW** at 130 K. (a) Coordination environment around the metal ions shown by 30 % probability thermal ellipsoids. (b) Crystal structure viewed along the *c*-axis, (c) *b*-axis, and (d) *a*-axis. Red, blue, pale green, grey, and black balls represent Rb, Co, W, C, and N atoms, respectively. Hydrogen atoms are omitted for clarity.

Table S5. Crystallographic data and structure refinement of the Co^{III}–W^{IV} phase of **RbCoW** at 130 K.

Compound	RbCoW (Co^{III}–W^{IV} phase)
Empirical formula	$C_{20}H_8CoN_{12}RbW$
Formula weight	744.63
Temperature/K	130(2)
Crystal system	triclinic
Space group	$P\bar{1}$
a/Å	7.2253(14)
b/Å	13.340(3)
c/Å	13.839(3)
α/°	118.671(8)
β/°	100.497(7)
γ/°	90.100(6)
V/Å^3	1144.6(4)
Z	2
ρ_{calc} /g cm^{-3}	2.161
μ/mm^{-1}	7.895
F(000)	700.0
Crystal size/mm^3	0.204 × 0.042 × 0.024
Radiation	MoKα (λ = 0.71075)
2θ range for data collection/°	5.978 to 54.952
Index ranges	$-9 \leq h \leq 9$<br>$-17 \leq k \leq 17$<br>$-15 \leq l \leq 17$
Reflections collected	10853
Independent reflections	5234 [R_{int} = 0.1070, R_{sigma} = 0.1500]
Data/restraints/parameters	5234/23/256
Goodness-of-fit on F^2	1.172
Final R indexes [I>=2σ (I)]	R_1 = 0.1105<br>wR_2 = 0.1690
Final R indexes [all data]	R_1 = 0.1424<br>wR_2 = 0.1797
Largest diff. peak/hole / e Å^{-3}	3.35/−5.70

Section 7. Comparison of the crystal structure of RbCoW between the Co^{II}–W^{V} phase and Co^{III}–W^{IV} phase

Table S6. Comparison of the bond lengths (W–C and Co–N) and bond angles (∠Co–N– C (3-CNpy)) of **RbCoW** between the Co^{II}–W^{V} phase (300 K) and Co^{III}–W^{IV} phase (130 K).

	Co^{II}–W^{V} phase	Co^{III}–W^{IV} phase
Temperature	300	130
W1–C1	2.139(13)	2.145(19)
W1–C2	2.129(14)	2.136(19)
W1–C3	2.135(13)	2.150(14)
W1–C4	2.144(13)	2.138(18)
W1–C5	2.173(19)	2.16(3)
W1–C6	2.153(14)	2.19(3)
W1–C7	2.146(16)	2.181(19)
W1–C8	2.155(18)	2.17(3)
Ave. W–C	2.15(13)	2.16(19)
Co1–N2	2.080(11)	1.878(14)
Co1–N3	2.092(12)	1.882(14)
Co1–N9	2.164(18)	2.00(5)
Co2–N1	2.084(11)	1.876(13)
Co2–N4	2.099(12)	1.902(14)
Co2–N11	2.14(3)	1.88(4)
Ave. Co–N	2.11(2)	1.90(3)
∠Co1 – N9 – C11(3-CNpy)	178.1(5)	174.8(5)
∠Co2 – N11 – C17(3-CNpy)	156.2(4)	163.2(5)

Table S7. List of atomic distances between the Rb ion and the surrounding atoms.

	Co^{II}–W^{V} phase	Co^{III}–W^{IV} phase
Rb–N5	3.146(19)	3.04(3)
Rb–C5	3.68(2)	3.55(4)
Rb–N7	3.14(2)	3.07(3)
Rb–C7	3.697(19)	3.58(3)
Rb–N12	2.989(18)	3.03(3)
Rb–C20	3.551(15)	3.55(3)
Rb–N5’	3.64(2)	3.62(3)
Rb–C5’	4.301(17)	4.34(3)
Rb–N6’	3.235(18)	3.20(3)
Rb–C6’	3.63(3)	3.60(4)
Rb–N7’	3.05(2)	2.947(18)
Rb–C7’	3.502(13)	3.468(18)

Section 8. First-principles electronic structure calculation of the Co^{III}–W^{IV} phase

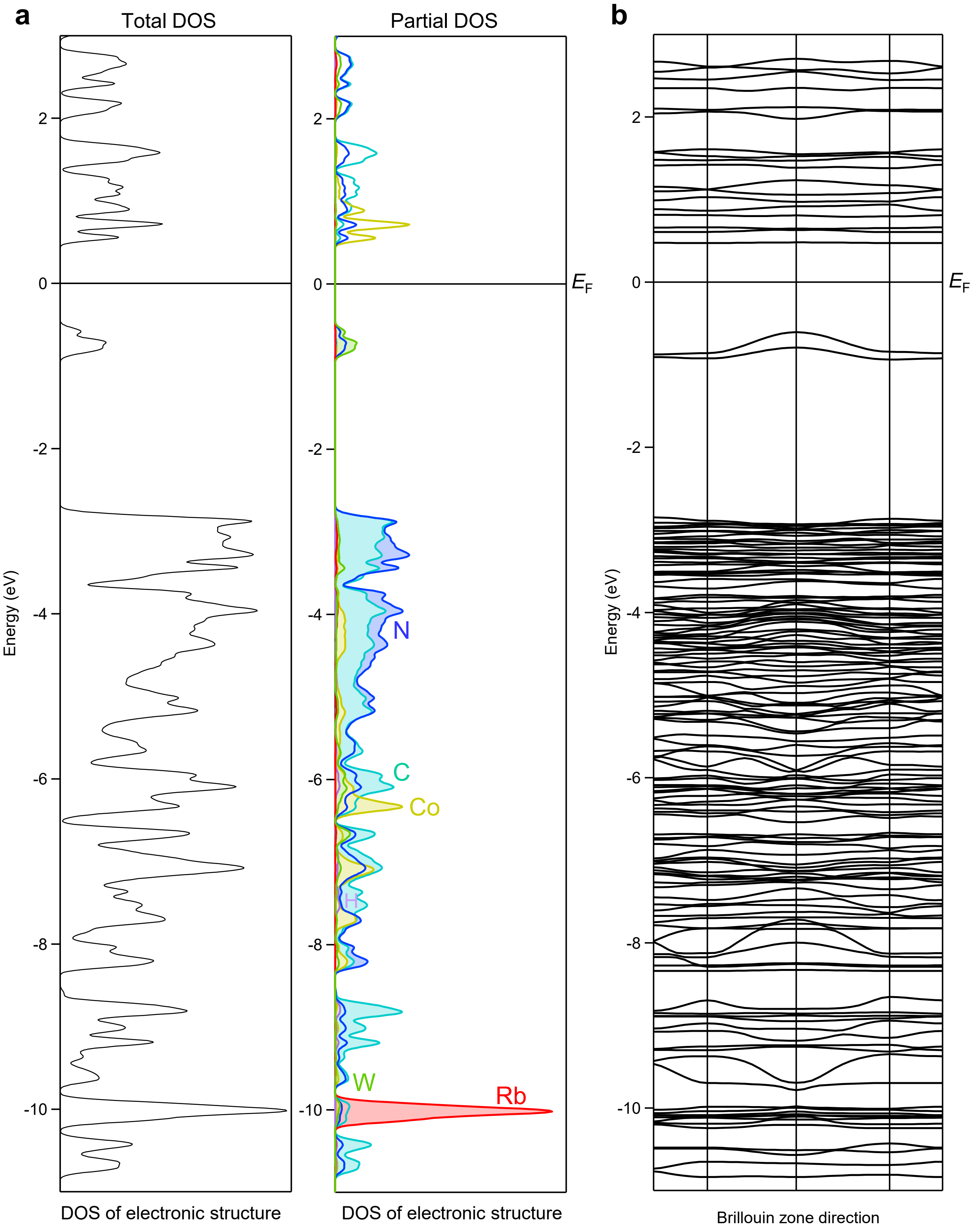

Figure S4. (a) Total DOS of the Co^{III}–W^{IV} phase (black line) and partial phonon DOS of Rb (red line), Co (dark yellow line), W (green line), C (light blue line), N (blue line), and H (purple line). (b) Band structure of the Co^{III}–W^{IV} phase of **RbCoW**.

Section 9. THz absorption spectrum of CsCoW

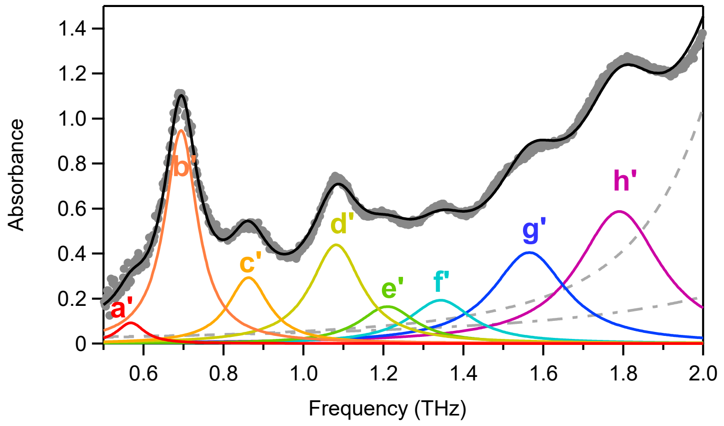

	a'	b'	c'	d'	e'	f'	g'	h'
Position (THz)	0.57	0.69	0.86	1.08	1.21	1.34	1.57	1.79
FWHM (THz)	0.08	0.10	0.12	0.16	0.17	0.19	0.23	0.25
Area	0.01	0.15	0.06	0.11	0.04	0.06	0.14	0.23

Figure S5. THz absorption spectrum of $Cs[Co^{II}(3\text{-cyanopyridine})_2][W^{V}(CN)_8]$ (**CsCoW**) with peaks at 0.57 (peak a'), 0.69 (peak b'), 0.86 (peak c'), 1.08 (peak d'), 1.21 (peak e'), 1.34 (peak f'), 1.57 (peak g'), and 1.79 THz (peak h'). Grey dots show the experimental data measured at room temperature, black line shows the fitted spectrum, and coloured lines indicate the components of each peak. Faint dashed grey lines represent the fitted curves of the higher frequency phonon modes.
